# Supplementary material for: Machine learning predictor PSPire screens for phase-separating proteins lacking intrinsically disordered regions
Source: Nat Commun. 2024 Mar 8;15:2147. doi: 10.1038/s41467-024-46445-y (PMC10923898; doi:10.1038/s41467-024-46445-y)
Supplement: Supplementary file 1 — Supplementary Information [file 41467_2024_46445_MOESM1_ESM.pdf]

# Machine learning predictor PSPire screens for phase-separating proteins lacking intrinsically disordered regions

Shuang Hou[1,6], Jiaojiao Hu[2,3,6], Zhaowei Yu[1], Dan Li[4,5], Cong Liu[2,3*], Yong Zhang[1*]

[1]State Key Laboratory of Cardiology and Medical Innovation Center, Institute for Regenerative Medicine, Department of Neurosurgery, Shanghai East Hospital, Shanghai Key Laboratory of Signaling and Disease Research, Frontier Science Center for Stem Cell Research, School of Life Sciences and Technology, Tongji University, Shanghai, 200092, China

[2]Interdisciplinary Research Center on Biology and Chemistry, Shanghai Institute of Organic Chemistry, Chinese Academy of Sciences, Shanghai, 201210, China

[3]State Key Laboratory of Chemical Biology, Shanghai Institute of Organic Chemistry, Chinese Academy of Sciences, Shanghai, 200032, China

[4]Bio-X Institutes, Key Laboratory for the Genetics of Developmental and Neuropsychiatric Disorders, Ministry of Education, Shanghai Jiao Tong University, Shanghai, 200240, China

[5]Zhangjiang Institute for Advanced Study, Shanghai Jiao Tong University, Shanghai, 200240, China

[6]These authors contributed equally: Shuang Hou, Jiaojiao Hu.

[*]Correspondence:

Yong Zhang, E-mail: yzhang@tongji.edu.cn

Cong Liu, E-mail: liulab@sioc.ac.cn

# Supplementary Figures

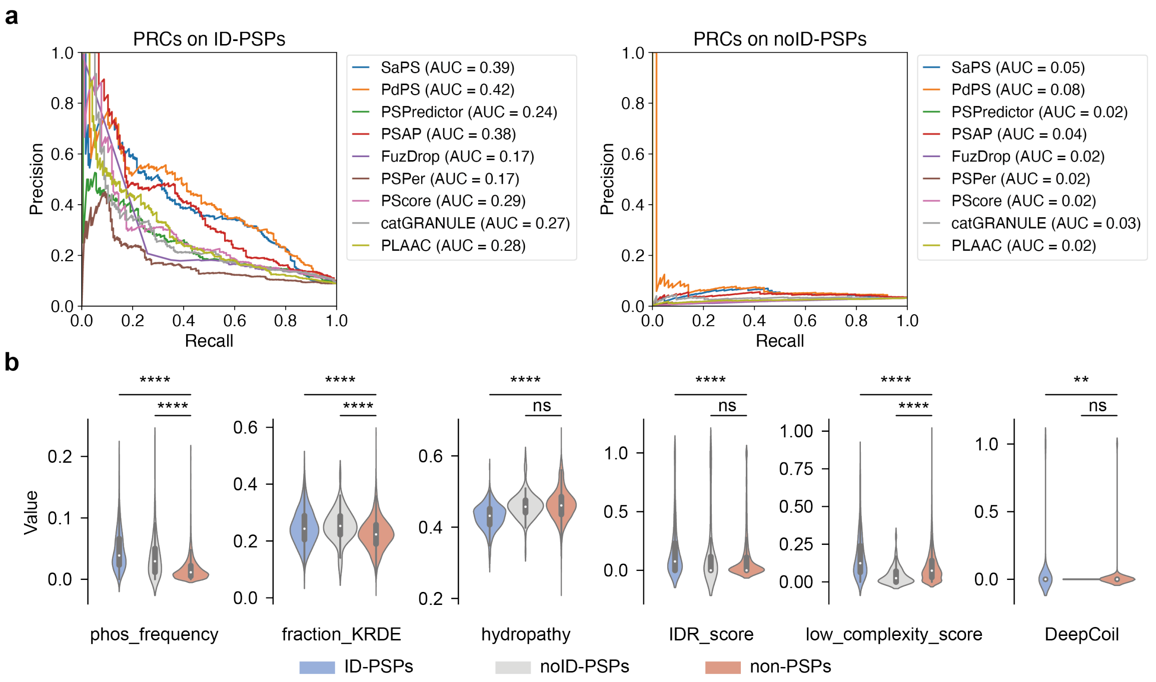


**Supplementary Figure 1. Performance of current PSP predictors on ID-PSPs and noID-PSPs. a,** Precision-recall curves (PRC) of eight predictors on the testing dataset. The performance was evaluated on ID-PSPs and noID-PSPs separately. The PhaSePred tool includes two models: SaPS for self-assembling proteins and PdPS for partner-dependent proteins. **b,** Comparison of six PS-related features that are used to train PhaSePred between the two types of PSPs (ID-PSPs and noID-PSPs) and non-PSPs. The six features calculated on the whole protein sequence are: phos_frequency (*i.e.*, the phosphorylation frequency), group_Charged (*i.e.*, the fraction of Charged group which includes K, R, D, and E), hydropathy (*i.e.*, the average hydropathy score), IDR score, low complexity score, and DeepCoil (*i.e.*, a binary score indicating the presence or absence of predicted structure). P-values were calculated using the two-sided Mann-Whitney U test: ns (not significant) for $p > 0.05$, ** for $p < 0.01$, and **** for $p < 0.0001$. The central dot indicates the median. The box represents interquartile range (IQR), 25–75th percentile. Whiskers extend to the data's minima and maxima within 1.5 * IQR. The comparison was conducted on the union of training and testing datasets which contained 389 ID-PSPs, 128 noID-PSPs, and 10,284 non-PSPs.

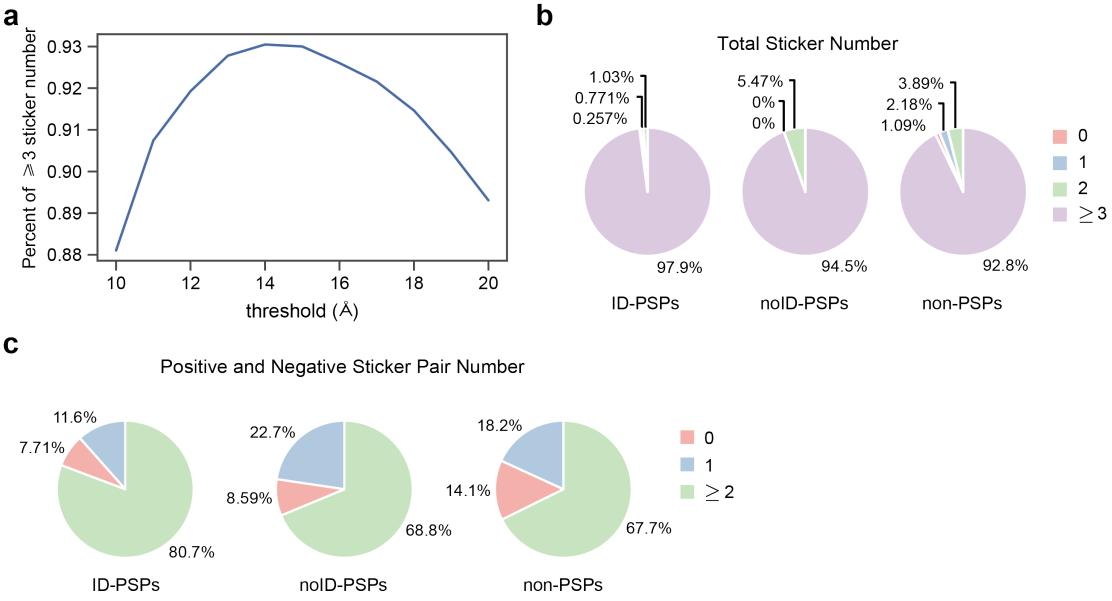


**Supplementary Figure 2. Calculation of sticker-related features for model construction. a,** Fraction of proteins with more than 3 stickers using a range of distances from 10 Å to 20 Å for the sticker calculation algorithm. **b and c,** Distribution of total sticker number and positive and negative sticker pair number for the three types of proteins (*i.e.*, ID-PSPs, noID-PSPs, and non-PSPs). The calculation was performed on the union of training and testing datasets which contained 389 ID-PSPs, 128 noID-PSPs, and 10,284 non-PSPs.

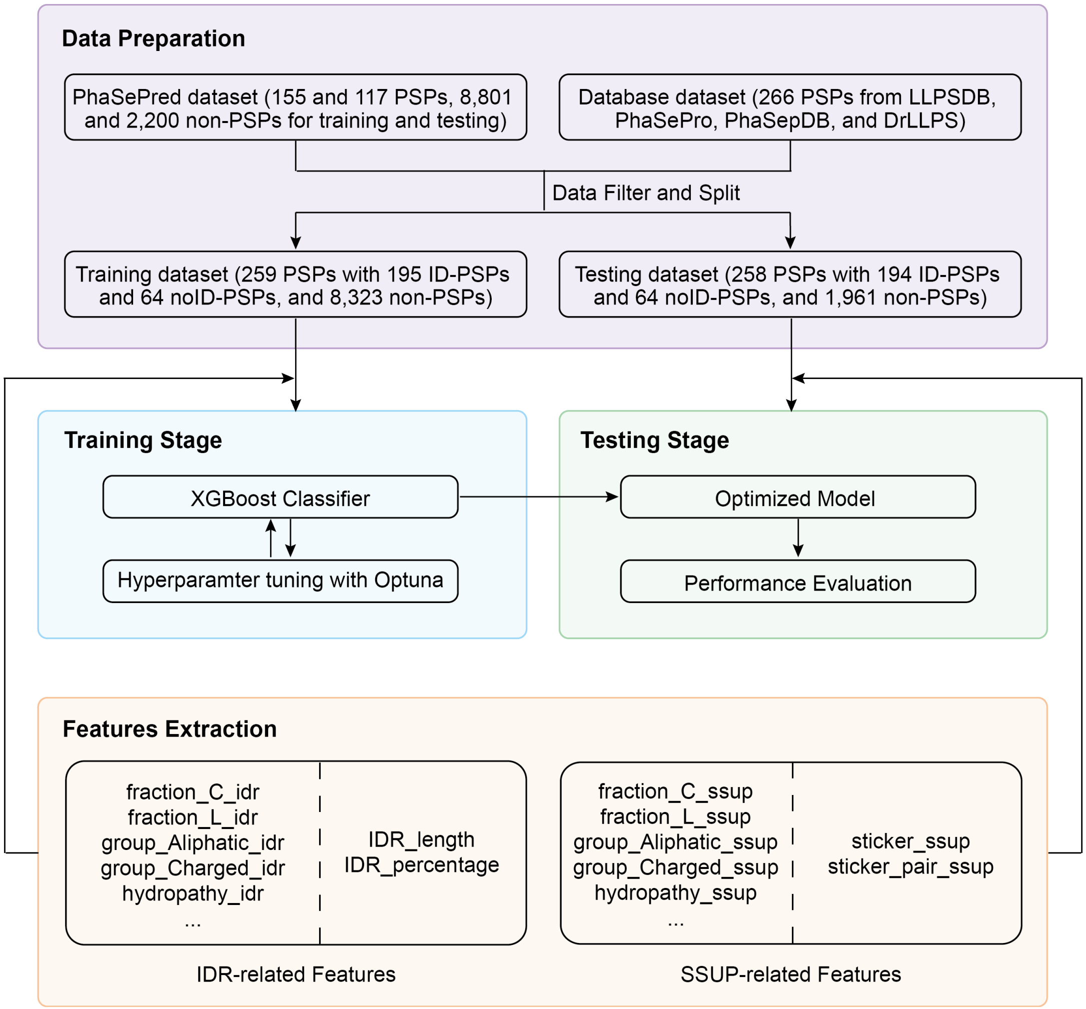


**Supplementary Figure 3. Overall framework of PSPire.** Two sources of phase-separating proteins (PSPs) were collected: (1) PSPs employed in the development of PhaSePred, which contained 155 PSPs and 8,801 non-PSPs for training, and 117 PSPs and 2,200 non-PSPs for testing; (2) 266 PSPs extracted from LLPSDB, PhaSePro, PhaSepDB, and DrLLPS databases. Proteins with a sequence length ≤ 100 or ≥ 2,700 amino acids were excluded. Additionally, to enable better comparison, proteins that could not be predicted by current predictors were also filtered out. The remaining proteins were then split into training and testing datasets. The training dataset comprised 259 PSPs (195 ID-PSPs and 64 noID-PSPs) and 8,323 non-PSPs, while the testing dataset consisted of 258 PSPs (194 ID-PSPs and 64 noID-PSPs) and 1,961 non-PSPs. Subsequently, IDR- and SSUP-related features were extracted for proteins in both datasets. During the training stage, the features of proteins in training dataset were utilized for model training. Meanwhile, hyperparameters for the XGBoost classifier were optimized using Optuna. The finalized, optimized model was then employed to predict the phase separation (PS) scores of proteins in the testing dataset, followed by a comprehensive performance evaluation.

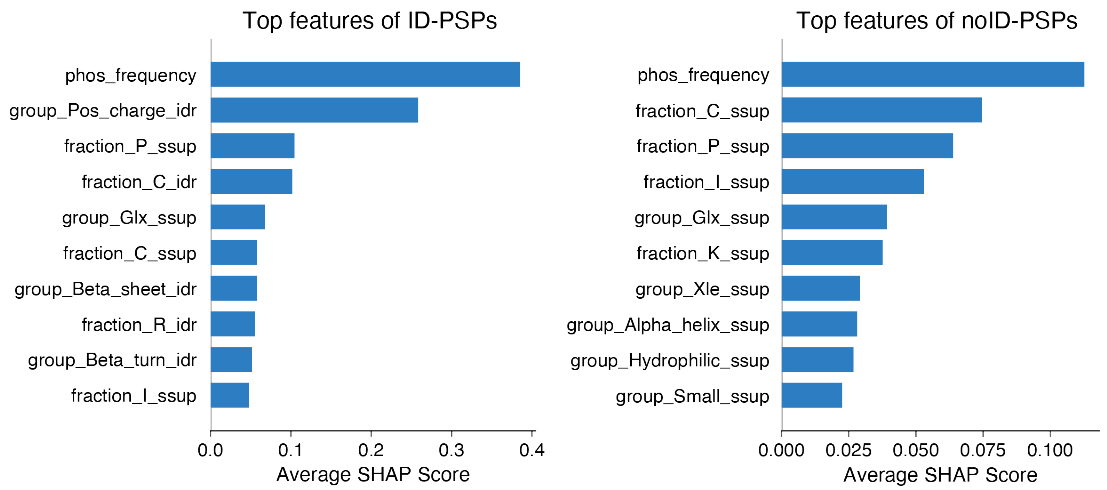


**Supplementary Figure 4. Features that attribute the top importance to PSPire prediction.** The importance was assessed using the average SHAP values, which were computed separately for differentiating between ID-PSPs and non-PSPs (left panel), and for distinguishing noID-PSPs from non-PSPs (right panel) in the testing dataset.

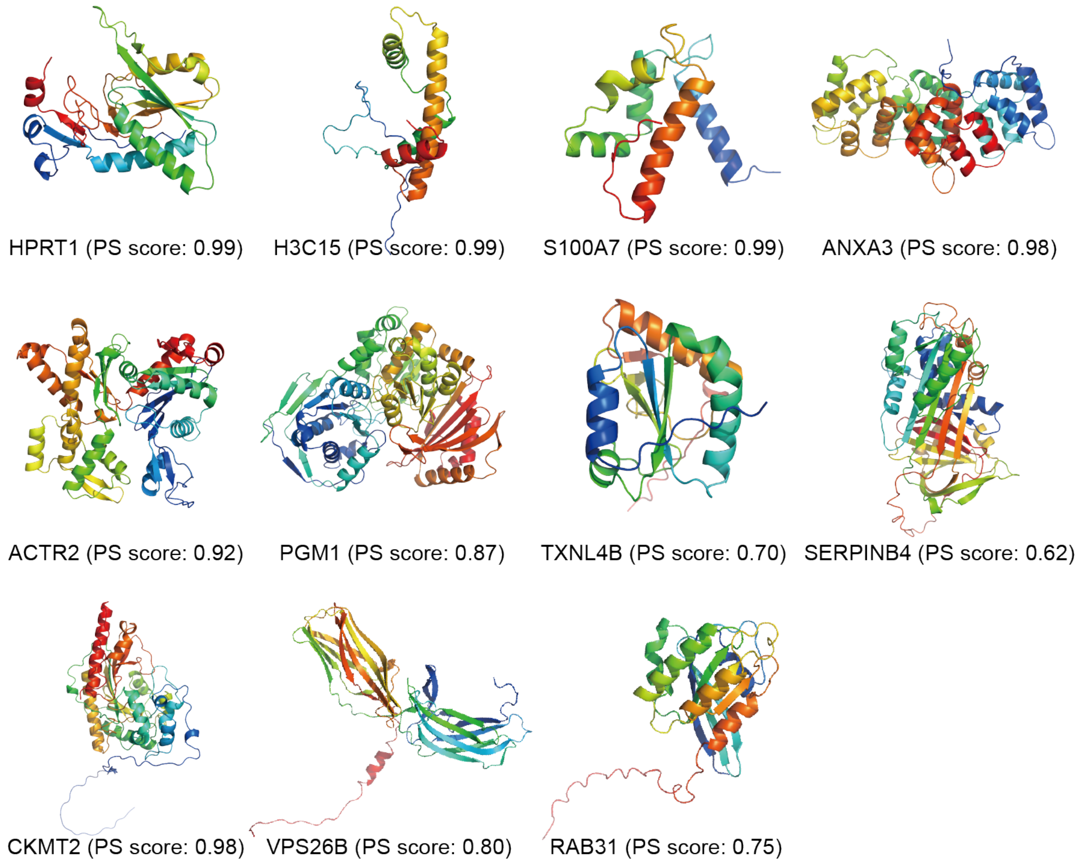


**Supplementary Figure 5. AlphaFold structures of eight candidate PSPs.** The PSPs are: HPRT1 (Uniprot ID: P00492), H3C15 (Uniprot ID: Q71DI3), S100A7 (UniProt ID: P31151), ANXA3 (UniProt ID: P12429), ACTR2 (Uniprot ID: P61160), PGM1 (UniProt ID: P36871), TXNL4B (UniProt ID: Q9NX01), SERPINB4 (UniProt ID: P48594), CKMT2 (UniProt ID: P17540), VPS26B (UniProt ID: Q4G0F5), and RAB31 (UniProt ID: Q13636). The PS scores for CKMT2, RAB31, and VPS26B were predicted by PSPire when IDR-related features were null.

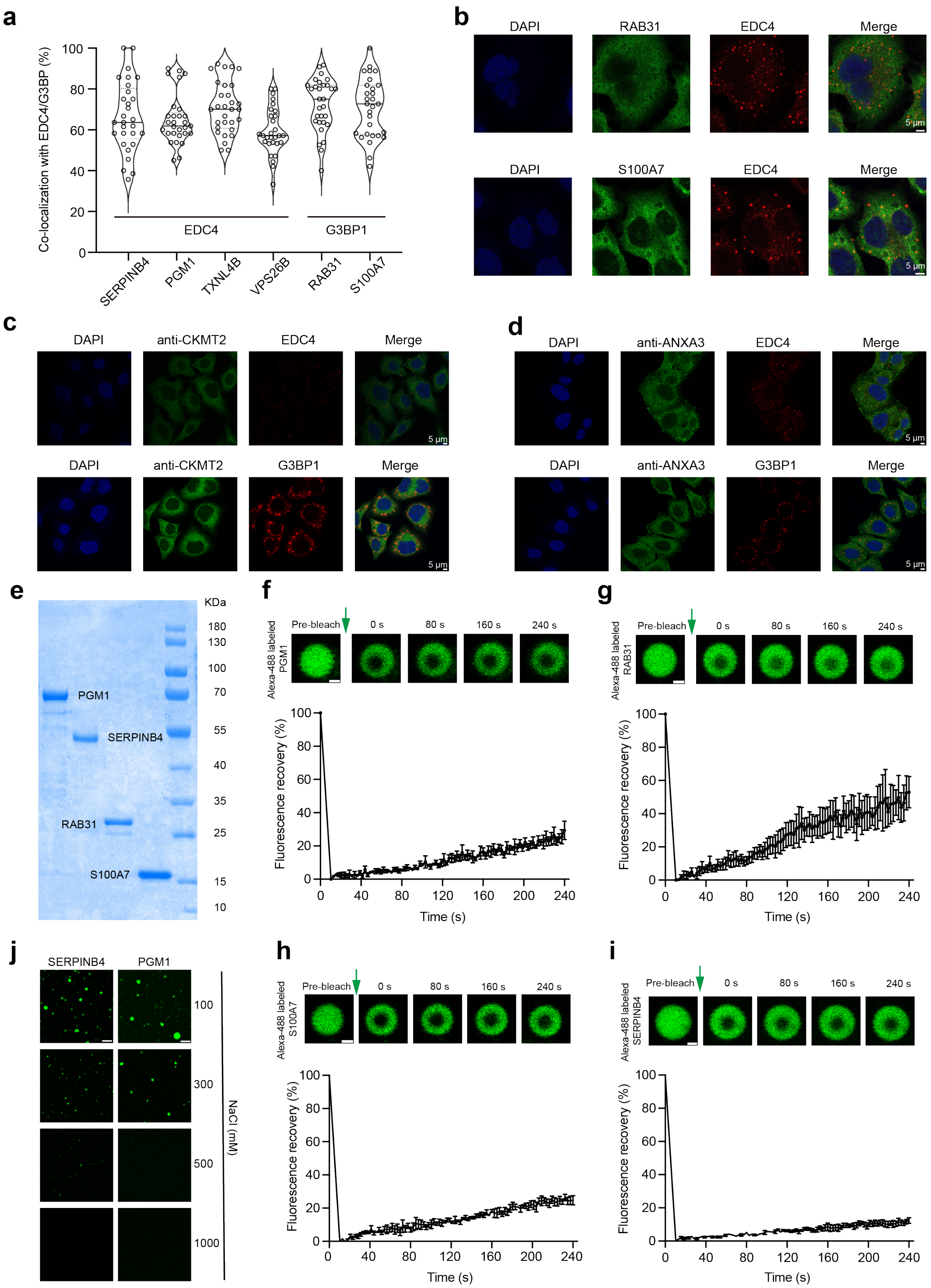


**Supplementary Figure 6. Validation of candidate PSPs predicted by PSPire. a,** Quantification of the co-localization percentage with EDC4/ G3BP1 signal. n=30. **b,** Immunostaining images of endogenous S100A7 and RAB31 in HeLa cells without stress. The S100A7 and RAB31 exhibit a diffuse pattern. **c,** Confocal microscopy images of

CKMT2 in HeLa cells without treatment (top panel) or treated with sodium arsenite (bottom panel). **d,** Confocal microscopy images of ANXA3 in HeLa cells in control conditions (top panel) and subjected to sodium arsenite-induced stress (bottom panel). **e**, Coomassie-stained denaturing gel of purified PGM1, SERPINB4, RAB31, and S100A7. **f-i,** The top panel presents confocal FRAP series for (f) PGM1, (g) RAB31, (h) S100A7, and (i) SERPINB4 in a buffer containing 20 mM Tris-HCl (pH 7.5), 150 mM NaCl, and 10% PEG 8000. The bottom panel shows corresponding fluorescence recovery over time for (f) PGM1, (g) RAB31, (h) S100A7, and (i) SERPINB4, post-photobleaching. Data shown are means ± SD, n = 3. Scale bars, 2 µm. **j,** Confocal images of 50 µM SERPINB4 and PGM1 in the addition of series concentrations of NaCl in buffer containing 50 mM Tris-HCl pH 7.5, 15% PEG 8000. Scale bars, 5 µm. The imaging was independently repeated 3 times with similar observations.

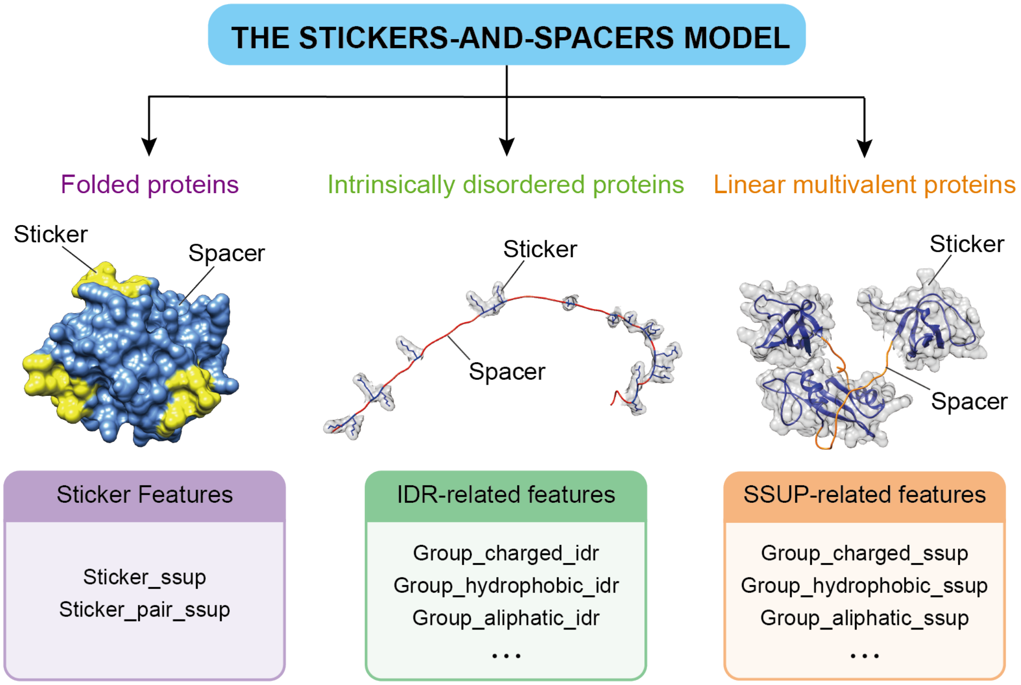


**Supplementary Figure 7. Schematic view of different types of stickers and spacers.** In folded proteins, stickers refer to interaction patches on the protein surface, whereas spacers comprise regions that do not participate in these interactions. For intrinsically disordered proteins, stickers can be individual amino acids, short linear motifs, or a combination of both, with spacers being the non-interacting residues interspersed among them. In the case of linear multivalent proteins, stickers are the binding sites on the surface of multiple folded domains, while spacers are the disordered linker regions and the surface residues not involved in binding. To accurately reflect the properties of different sticker types, sticker-related, IDR-related, and SSUP-related features were calculated for each of the three protein categories individually.

## Supplementary Tables

Supplementary Table 1. Details of current PSP predictors used for comparison with PSPire.

| Predictor | Description | Information used for prediction | Availability | Year | Ref. |
|---|---|---|---|---|---|
| PhaSePred | Metapredictor for self-assembling proteins and partner-dependent proteins | Multiple PS-related features as well as the prediction scores from several PSP predictors | predict.phasep.pro | 2022 | 26 |
| PSPredictor | Sequence-based prediction tool | Protein sequence embedding | www.pkumdl.cn/PSPredictor | 2022 | 25 |
| PSAP | Machine-learning classifier based on amino acid content | Amino acid features | github.com/Guido497/phase-separation | 2021 | 24 |
| FuzDrop | Prediction of protein droplet-promoting propensity | Variables based on sequence | doi.org/10.1073/pnas.2007670117 | 2020 | 23 |
| PSPer | Prediction of prion-like RNA-binding proteins | Residue-level and domain-level characteristics derived from sequence | www.bio2byte.be/b2btools/psp/ | 2019 | 18 |
| PScore | Predictor of proteins with pi-pi interactions | Pi-pi contact frequencies | elifesciences.org/articles/31486 | 2018 | 17 |
| catGRANULE | Prediction of granule-formation propensity | RNA binding and disordered propensities, amino acid patterns, sequence length | www.tartaglialab.com | 2016 | 16 |
| PLAAC | Prediction of proteins with prion-like amino acid composition | Amino acid frequencies in prion-like domain of *S.cerevisiae* | plaac.wi.mit.edu | 2014 | 15 |

Supplementary Table 2. Evaluation of PSPire and eight predictors on six datasets: the testing dataset, the G3BP1 proximity labelling set, the DACT1-particulate proteome set, the RNAgranuleDB Tier1 set, the PhaSepDB low and high throughput MLO set, and the DrLLPS MLO set. The PhaSePred tool includes two models: SaPS for self-assembling proteins and PdPS for partner-dependent proteins. The evaluation metrics include Matthews correlation coefficient (MCC), F1-score, sensitivity, specificity, accuracy, false positive rate (FPR), and false negative rate (FNR). The best results for each row are marked in bold.

| PSPs | Dataset | Type | PSPire | SaPS | PdPS | PSPredictor | PSAP | FuzDrop | PSPer | PScore | catGRANULE | PLAAC |
|---|---|---|---|---|---|---|---|---|---|---|---|---|
| noID-PSPs | Testing dataset | MCC | **0.22** | 0.08 | 0.10 | -0.07 | 0.07 | -0.11 | -0.06 | -0.06 | 0.02 | -0.04 |
| | | F1-score | **0.18** | 0.09 | 0.10 | 0.05 | 0.09 | 0.04 | 0.04 | 0.05 | 0.07 | 0.04 |
| | | Sensitivity | **0.73** | 0.58 | 0.64 | 0.61 | 0.61 | 0.55 | 0.42 | 0.63 | 0.52 | 0.38 |
| | | Specificity | **0.79** | 0.64 | 0.63 | 0.22 | 0.60 | 0.20 | 0.42 | 0.23 | 0.54 | 0.50 |
| | | Accuracy | **0.79** | 0.64 | 0.63 | 0.24 | 0.60 | 0.21 | 0.42 | 0.24 | 0.54 | 0.49 |
| | | FPR | **0.21** | 0.36 | 0.37 | 0.78 | 0.40 | 0.80 | 0.58 | 0.77 | 0.46 | 0.50 |
| | | FNR | **0.27** | 0.42 | 0.36 | 0.39 | 0.39 | 0.45 | 0.58 | 0.38 | 0.48 | 0.63 |
| | G3BP1 proximity labelling | MCC | **0.40** | 0.30 | 0.23 | -0.08 | 0.14 | -0.12 | -0.10 | -0.05 | 0.14 | -0.08 |
| | | F1-score | **0.36** | 0.28 | 0.22 | 0.08 | 0.16 | 0.07 | 0.06 | 0.08 | 0.15 | 0.07 |
| | | Sensitivity | **0.90** | 0.80 | 0.78 | 0.77 | 0.65 | 0.56 | 0.42 | 0.68 | 0.72 | 0.53 |
| | | Specificity | **0.83** | 0.79 | 0.71 | 0.11 | 0.65 | 0.22 | 0.37 | 0.22 | 0.59 | 0.30 |
| | | Accuracy | **0.83** | 0.79 | 0.71 | 0.14 | 0.65 | 0.23 | 0.37 | 0.24 | 0.59 | 0.31 |
| | | FPR | **0.17** | 0.21 | 0.29 | 0.89 | 0.35 | 0.78 | 0.63 | 0.78 | 0.41 | 0.70 |
| | | FNR | **0.10** | 0.20 | 0.22 | 0.23 | 0.35 | 0.44 | 0.58 | 0.32 | 0.28 | 0.47 |
| | DACT1-particulate proteome | MCC | **0.48** | 0.26 | 0.25 | -0.12 | 0.17 | -0.11 | -0.03 | -0.07 | 0.07 | -0.13 |
| | | F1-score | **0.48** | 0.26 | 0.25 | 0.05 | 0.19 | 0.09 | 0.10 | 0.09 | 0.14 | 0.07 |
| | | Sensitivity | **0.84** | 0.76 | 0.80 | 0.23 | 0.72 | 0.59 | 0.48 | 0.59 | 0.62 | 0.40 |
| | | Specificity | **0.89** | 0.74 | 0.70 | 0.51 | 0.62 | 0.22 | 0.46 | 0.28 | 0.53 | 0.35 |
| | | Accuracy | **0.89** | 0.74 | 0.70 | 0.49 | 0.62 | 0.24 | 0.46 | 0.30 | 0.54 | 0.35 |
| | | FPR | **0.11** | 0.26 | 0.30 | 0.49 | 0.38 | 0.78 | 0.54 | 0.72 | 0.47 | 0.65 |
| | | FNR | **0.16** | 0.24 | 0.20 | 0.77 | 0.28 | 0.41 | 0.52 | 0.41 | 0.38 | 0.60 |
| | RNAgranuleDB | MCC | **0.24** | 0.08 | 0.10 | -0.06 | 0.08 | -0.05 | -0.04 | -0.03 | 0.04 | -0.04 |
| | | F1-score | **0.17** | 0.09 | 0.09 | 0.03 | 0.07 | 0.04 | 0.04 | 0.04 | 0.06 | 0.04 |
| | | Sensitivity | **0.86** | 0.51 | 0.61 | 0.33 | 0.71 | 0.61 | 0.45 | 0.71 | 0.59 | 0.35 |
| | | Specificity | **0.78** | 0.73 | 0.68 | 0.47 | 0.55 | 0.24 | 0.43 | 0.22 | 0.53 | 0.51 |
| | | Accuracy | **0.78** | 0.72 | 0.67 | 0.46 | 0.55 | 0.25 | 0.43 | 0.23 | 0.53 | 0.51 |
| | | FPR | **0.22** | 0.27 | 0.32 | 0.53 | 0.45 | 0.76 | 0.57 | 0.78 | 0.47 | 0.49 |
| | | FNR | **0.14** | 0.49 | 0.39 | 0.67 | 0.29 | 0.39 | 0.55 | 0.29 | 0.41 | 0.65 |
| | PhaSepDB_MLO | MCC | **0.44** | 0.15 | 0.22 | -0.27 | 0.14 | -0.23 | -0.11 | -0.20 | -0.01 | -0.25 |
| | | F1-score | **0.65** | 0.45 | 0.52 | 0.33 | 0.48 | 0.38 | 0.41 | 0.38 | 0.41 | 0.31 |
| | | Sensitivity | **0.75** | 0.47 | 0.61 | 0.47 | 0.58 | 0.59 | 0.59 | 0.56 | 0.52 | 0.42 |
| | | Specificity | **0.72** | 0.69 | 0.63 | 0.27 | 0.56 | 0.19 | 0.30 | 0.24 | 0.48 | 0.32 |
| | | Accuracy | **0.73** | 0.61 | 0.62 | 0.33 | 0.57 | 0.33 | 0.40 | 0.35 | 0.49 | 0.35 |
| | | FPR | **0.28** | 0.31 | 0.37 | 0.73 | 0.44 | 0.81 | 0.70 | 0.76 | 0.52 | 0.68 |
| | | FNR | **0.25** | 0.53 | 0.39 | 0.53 | 0.42 | 0.41 | 0.41 | 0.44 | 0.48 | 0.58 |
| | DrLLPS_MLO | MCC | **0.53** | 0.21 | 0.27 | -0.29 | 0.22 | -0.22 | -0.12 | -0.16 | 0.04 | -0.19 |
| | | F1-score | **0.69** | 0.48 | 0.53 | 0.31 | 0.52 | 0.36 | 0.38 | 0.38 | 0.43 | 0.37 |
| | | Sensitivity | **0.77** | 0.54 | 0.66 | 0.48 | 0.70 | 0.60 | 0.59 | 0.65 | 0.61 | 0.60 |
| | | Specificity | **0.79** | 0.69 | 0.63 | 0.23 | 0.54 | 0.20 | 0.29 | 0.21 | 0.44 | 0.22 |
| | | Accuracy | **0.78** | 0.64 | 0.64 | 0.31 | 0.59 | 0.32 | 0.39 | 0.35 | 0.49 | 0.34 |
| | | FPR | **0.21** | 0.31 | 0.37 | 0.77 | 0.46 | 0.80 | 0.71 | 0.79 | 0.56 | 0.78 |
| | | FNR | **0.23** | 0.46 | 0.34 | 0.52 | 0.30 | 0.40 | 0.41 | 0.35 | 0.39 | 0.40 |
| ID-PSPs | Testing dataset | MCC | **0.40** | 0.34 | 0.38 | 0.20 | 0.25 | 0.18 | 0.11 | 0.21 | 0.19 | 0.20 |
| | | F1-score | **0.43** | 0.37 | 0.41 | 0.26 | 0.30 | 0.25 | 0.21 | 0.27 | 0.26 | 0.26 |
| | | Sensitivity | **0.77** | **0.77** | 0.75 | 0.68 | 0.75 | 0.66 | 0.56 | 0.65 | 0.69 | 0.66 |
| | | Specificity | **0.82** | 0.76 | 0.81 | 0.65 | 0.67 | 0.65 | 0.63 | 0.69 | 0.63 | 0.66 |
| | | Accuracy | **0.82** | 0.76 | 0.81 | 0.65 | 0.68 | 0.65 | 0.63 | 0.69 | 0.64 | 0.66 |
| | | FPR | **0.18** | 0.24 | 0.19 | 0.35 | 0.33 | 0.35 | 0.37 | 0.31 | 0.37 | 0.34 |
| | | FNR | **0.23** | **0.23** | 0.25 | 0.32 | 0.25 | 0.34 | 0.44 | 0.35 | 0.31 | 0.34 |
| | G3BP1 proximity labelling | MCC | **0.43** | 0.35 | 0.30 | 0.12 | 0.26 | 0.05 | 0.08 | 0.05 | 0.20 | 0.07 |
| | | F1-score | **0.42** | 0.35 | 0.30 | 0.17 | 0.28 | 0.14 | 0.16 | 0.14 | 0.23 | 0.15 |
| | | Sensitivity | **0.86** | 0.80 | 0.83 | 0.71 | 0.69 | 0.62 | 0.56 | 0.60 | 0.69 | 0.47 |
| | | Specificity | **0.84** | 0.80 | 0.73 | 0.53 | 0.77 | 0.48 | 0.60 | 0.51 | 0.69 | 0.66 |
| | | Accuracy | **0.84** | 0.80 | 0.74 | 0.55 | 0.76 | 0.48 | 0.60 | 0.51 | 0.69 | 0.65 |
| | | FPR | **0.16** | 0.20 | 0.27 | 0.47 | 0.23 | 0.52 | 0.40 | 0.49 | 0.31 | 0.34 |
| | | FNR | **0.14** | 0.20 | 0.17 | 0.29 | 0.31 | 0.38 | 0.44 | 0.40 | 0.31 | 0.53 |
| | DACT1-particulate proteome | MCC | **0.32** | 0.26 | 0.29 | 0.06 | 0.22 | 0.06 | 0.10 | 0.05 | 0.15 | 0.00 |
| | | F1-score | **0.29** | 0.24 | 0.26 | 0.11 | 0.21 | 0.11 | 0.13 | 0.10 | 0.15 | 0.08 |
| | | Sensitivity | **0.77** | **0.77** | 0.76 | 0.63 | 0.72 | 0.65 | 0.61 | 0.64 | 0.73 | 0.44 |
| | | Specificity | **0.83** | 0.77 | 0.81 | 0.53 | 0.75 | 0.50 | 0.62 | 0.48 | 0.61 | 0.56 |
| | | Accuracy | **0.83** | 0.77 | 0.81 | 0.53 | 0.75 | 0.51 | 0.62 | 0.49 | 0.62 | 0.55 |
| | | FPR | **0.17** | 0.23 | 0.19 | 0.47 | 0.25 | 0.50 | 0.38 | 0.52 | 0.39 | 0.44 |
| | | FNR | **0.23** | **0.23** | 0.24 | 0.37 | 0.28 | 0.35 | 0.39 | 0.36 | 0.27 | 0.56 |
| | RNAgranuleDB | MCC | **0.37** | 0.36 | 0.35 | 0.24 | 0.29 | 0.20 | 0.18 | 0.24 | 0.29 | 0.25 |
| | | F1-score | **0.44** | 0.42 | 0.42 | 0.33 | 0.36 | 0.30 | 0.29 | 0.32 | 0.36 | 0.34 |
| | | Sensitivity | 0.73 | **0.77** | 0.70 | 0.67 | 0.74 | 0.72 | 0.65 | 0.75 | 0.76 | 0.68 |
| | | Specificity | **0.79** | 0.75 | 0.78 | 0.68 | 0.69 | 0.59 | 0.62 | 0.61 | 0.68 | 0.69 |
| | | Accuracy | **0.78** | 0.75 | 0.77 | 0.68 | 0.69 | 0.61 | 0.62 | 0.63 | 0.69 | 0.69 |
| | | FPR | **0.21** | 0.25 | 0.22 | 0.32 | 0.31 | 0.41 | 0.38 | 0.39 | 0.32 | 0.31 |
| | | FNR | 0.27 | **0.23** | 0.30 | 0.33 | 0.26 | 0.28 | 0.35 | 0.25 | 0.24 | 0.32 |
| | PhaSepDB_MLO | MCC | 0.31 | 0.34 | **0.35** | 0.21 | 0.27 | 0.20 | 0.16 | 0.17 | 0.28 | 0.12 |
| | | F1-score | 0.67 | 0.67 | **0.71** | 0.66 | 0.69 | 0.67 | 0.61 | 0.65 | 0.69 | 0.60 |
| | | Sensitivity | 0.61 | 0.60 | **0.68** | 0.63 | **0.68** | 0.66 | 0.56 | 0.63 | **0.68** | 0.56 |
| | | Specificity | 0.71 | **0.74** | 0.67 | 0.58 | 0.59 | 0.55 | 0.60 | 0.54 | 0.60 | 0.56 |
| | | Accuracy | 0.65 | 0.66 | **0.68** | 0.61 | 0.64 | 0.61 | 0.58 | 0.59 | 0.65 | 0.56 |
| | | FPR | 0.29 | **0.26** | 0.33 | 0.42 | 0.41 | 0.45 | 0.40 | 0.46 | 0.40 | 0.44 |
| | | FNR | 0.39 | 0.40 | **0.32** | 0.37 | **0.32** | 0.34 | 0.44 | 0.37 | **0.32** | 0.44 |
| | DrLLPS_MLO | MCC | 0.37 | 0.36 | **0.39** | 0.22 | 0.31 | 0.19 | 0.11 | 0.18 | 0.31 | 0.13 |
| | | F1-score | 0.69 | 0.68 | **0.72** | 0.64 | 0.67 | 0.63 | 0.57 | 0.62 | 0.69 | 0.59 |
| | | Sensitivity | 0.65 | 0.63 | **0.72** | 0.65 | 0.66 | 0.64 | 0.54 | 0.63 | 0.71 | 0.57 |
| | | Specificity | 0.72 | **0.73** | 0.67 | 0.56 | 0.65 | 0.54 | 0.57 | 0.54 | 0.60 | 0.56 |
| | | Accuracy | 0.68 | 0.68 | **0.70** | 0.61 | 0.65 | 0.60 | 0.56 | 0.59 | 0.66 | 0.57 |
| | | FPR | 0.28 | **0.27** | 0.33 | 0.44 | 0.35 | 0.46 | 0.43 | 0.46 | 0.40 | 0.44 |
| | | FNR | 0.35 | 0.37 | **0.28** | 0.35 | 0.34 | 0.36 | 0.46 | 0.37 | 0.29 | 0.43 |

Supplementary Table 3. Hyperparameters used to tune XGBoost classifier.

| **Hyperparameters** | **Description** | **Data range** | **Search Range** |
|---|---|---|---|
| learning_rate | The learning rate. | [0,1] | [0.01,0.3] |
| n_estimators | The number of trees. | [1,∞] | [50,1000] |
| max_depth | The maximum depth of a tree. | [0,∞] | [3,10] |
| min_child_weight | The minimum sum of instance weight needed in a child. | [0,∞] | [1,10] |
| subsample | The subsample ratio of the training instances for each tree. | (0,1] | [0.5,1] |
| colsample_bytree | The subsample ratio of features when constructing each tree. | (0,1] | [0.5,1] |
| gamma | The minimum loss reduction required to make a further split on a leaf node of the tree. | [0,∞] | [0,1] |
| reg_lambda | L2 regularization term on weights. | [0,∞] | [1e-5,100] |
| reg_alpha | L1 regularization term on weights. | [0,∞] | [1e-5,100] |

Supplementary Table 4. Optimized values of XGBoost hyperparameters for the model with and without the Phos frequency feature (*i.e.*, human model and other species model).

| **Hyperparameters** | **Optimal value** | |
|---|---|---|
| | **Human Model** | **Other Species Model** |
| learning_rate | 0.1311 | 0.1202 |
| n_estimators | 811 | 971 |
| max_depth | 7 | 7 |
| min_child_weight | 3 | 1 |
| subsample | 0.9620 | 0.9215 |
| colsample_bytree | 0.9814 | 0.8331 |
| gamma | 0.3062 | 0.0003 |
| reg_lambda | 1.1258 | 2.6413 |
| reg_alpha | 0.0289 | 0.0052 |
