# Supplementary material for: Machine learning predictor PSPire screens for phase-separating proteins lacking intrinsically disordered regions
Source: Nat Commun. 2024 Mar 8;15:2147. doi: 10.1038/s41467-024-46445-y (PMC10923898; doi:10.1038/s41467-024-46445-y)
Supplement: Supplementary file 2 — Reporting Summary [file 41467_2024_46445_MOESM2_ESM.pdf]

nature portfolio

Corresponding author(s): Yong Zhang
Last updated by author(s): Feb 15, 2024

Reporting Summary

Nature Portfolio wishes to improve the reproducibility of the work that we publish. This form provides structure for consistency and transparency in reporting. For further information on Nature Portfolio policies, see our Editorial Policies and the Editorial Policy Checklist.

Statistics

For all statistical analyses, confirm that the following items are present in the figure legend, table legend, main text, or Methods section.

n/a	Confirmed	
☐	☒	The exact sample size (*n*) for each experimental group/condition, given as a discrete number and unit of measurement
☐	☒	A statement on whether measurements were taken from distinct samples or whether the same sample was measured repeatedly
☐	☒	The statistical test(s) used AND whether they are one- or two-sided<br>*Only common tests should be described solely by name; describe more complex techniques in the Methods section.*
☒	☐	A description of all covariates tested
☐	☒	A description of any assumptions or corrections, such as tests of normality and adjustment for multiple comparisons
☐	☒	A full description of the statistical parameters including central tendency (e.g. means) or other basic estimates (e.g. regression coefficient) AND variation (e.g. standard deviation) or associated estimates of uncertainty (e.g. confidence intervals)
☐	☒	For null hypothesis testing, the test statistic (e.g. *F*, *t*, *r*) with confidence intervals, effect sizes, degrees of freedom and *P* value noted<br>*Give P values as exact values whenever suitable.*
☒	☐	For Bayesian analysis, information on the choice of priors and Markov chain Monte Carlo settings
☒	☐	For hierarchical and complex designs, identification of the appropriate level for tests and full reporting of outcomes
☒	☐	Estimates of effect sizes (e.g. Cohen's *d*, Pearson's *r*), indicating how they were calculated

Our web collection on statistics for biologists contains articles on many of the points above.

Software and code

Policy information about availability of computer code

Data collection	LAS X (Leica) was used for confocal image collection.
Data analysis	The following softwares were used for data analysis: MobiDB-lite (v3.8.4) and PSAIA (v1.0). And analysis in R and Python used the following packages: DSSP (v2.2.1), PyMOL (v2.5.0), UCSF Chimera (v1.16), XGBoost (v1.6.2), biopython (v1.79), scikit-learn (v1.1.2), numpy (v1.23.3), pandas (v1.4.1), Image Lab (v3.0), Image J (v2.0.3) and R (v4.1.1). Scripts generated within this study are available at Github (https://github.com/TongjiZhanglab/PSPire).

For manuscripts utilizing custom algorithms or software that are central to the research but not yet described in published literature, software must be made available to editors and reviewers. We strongly encourage code deposition in a community repository (e.g. GitHub). See the Nature Portfolio guidelines for submitting code & software for further information.

Data

Policy information about availability of data

All manuscripts must include a data availability statement. This statement should provide the following information, where applicable:

- Accession codes, unique identifiers, or web links for publicly available datasets
- A description of any restrictions on data availability
- For clinical datasets or third party data, please ensure that the statement adheres to our policy

The PDB format files of human protein structures (identifier: UP000005640) can be downloaded from the AlphaFold DB website (https://ftp.ebi.ac.uk/pub/databases/alphafold/latest/UP000005640_9606_HUMAN_v4.tar). All datasets used in this study are publicly available and detailed in Supplementary Data 4 and

Supplementary Data 5. The following four databases were used for the collection of phase-separating protein datasets: LLPSDB (http://bio-comp.org.cn/llpsdb), PhaSePro (https://phasepro.elte.hu), PhaSepDB (http://db.phasep.pro), and DrLLPS (https://llps.biocuckoo.cn). Pre-calculated PSPire predicted scores and residue positions in structured superficial regions (SSUP) and sticker regions for proteins in the following model organism proteomes generated in this study have been deposited in the GitHub repository (https://github.com/TongjiZhanglab/PSPire): Arabidopsis thaliana, Caenorhabditis elegans, Candida albicans, Danio rerio, Dictyostelium discoideum, Drosophila melanogaster, Escherichia coli, Glycine max, Homo sapiens, Methanocaldococcus jannaschii, Mus musculus, Oryza sativa, Rattus norvegicus, Saccharomyces cerevisiae, Schizosaccharomyces pombe, and Zea mays. The secondary structure states and relative surface exposure data of proteins in human proteome generated in this study could also be downloaded from the GitHub repository. Source data are provided with this paper.

Research involving human participants, their data, or biological material

Policy information about studies with human participants or human data. See also policy information about sex, gender (identity/presentation), and sexual orientation and race, ethnicity and racism.

Reporting on sex and gender	n/a
Reporting on race, ethnicity, or other socially relevant groupings	n/a
Population characteristics	n/a
Recruitment	n/a
Ethics oversight	n/a

Note that full information on the approval of the study protocol must also be provided in the manuscript.

Field-specific reporting

Please select the one below that is the best fit for your research. If you are not sure, read the appropriate sections before making your selection.

☒ Life sciences ☐ Behavioural & social sciences ☐ Ecological, evolutionary & environmental sciences

For a reference copy of the document with all sections, see nature.com/documents/nr-reporting-summary-flat.pdf

Life sciences study design

All studies must disclose on these points even when the disclosure is negative.

Sample size	No statistical methods were used to predetermine sample size. For better comparison with previous phase-separating protein predictors, similar sample sizes were used as previous related study (PMID: 35687670), which are generally sufficient for statistical test.
Data exclusions	Since proteins longer than 2,700 amino acids were segmented into overlapping fragments by AlphaFold, proteins with a sequence length ≥ 2,700 amino acids were filtered out. To ensure robustness, proteins with a sequence length ≤ 100 amino acids were also filtered out. For comparison with other prediction tools, proteins that cannot be predicted by these tools were also filtered out.
Replication	At least three independent biological repeats were performed for the biochemical experiments. All attempts at replication were successful.
Randomization	The datasets were randomly divided into training and testing datasets. A random seed of 42 was used consistently throughout the process to ensure reproducibility.
Blinding	The investigators responsible for images collection and data analysis were blinded to group allocations.

Reporting for specific materials, systems and methods

We require information from authors about some types of materials, experimental systems and methods used in many studies. Here, indicate whether each material, system or method listed is relevant to your study. If you are not sure if a list item applies to your research, read the appropriate section before selecting a response.

Materials & experimental systems

n/a	Involved in the study	
☐	☒	Antibodies
☐	☒	Eukaryotic cell lines
☒	☐	Palaeontology and archaeology
☒	☐	Animals and other organisms
☒	☐	Clinical data
☒	☐	Dual use research of concern
☒	☐	Plants

Methods

n/a	Involved in the study	
☒	☐	ChIP-seq
☒	☐	Flow cytometry
☒	☐	MRI-based neuroimaging

Antibodies

Antibodies used	Primary antibodies: anti-PGM1 (abs117064, absin), anti-SERPINB4 (abs134793, absin), anti-S100A7 (abs139303, absin), anti-TXNL4B (abs117670, absin), anti-RAB31 (abs134703, absin), anti-VPS26B (absin134908, absin), anti-G3BP1 (611127, BD Biosciences,), anti-EDC4 (sc-376382, Santa Cruz Biotechnology).<br>Secondary antibodies: Goat anti-rabbit-Alexa Flour 488 (Invitrogen, A-11008), goat anti-mouse-Alexa Flour 568 (Invitrogen, A-11004).
Validation	Anti-PGM1 antibody has been validated by Absin in immunostaining of U2OS cells. Anti-TXNL4B antibody has been validated by Absin in immunofluorescence analysis of A549 cells. Anti-RAB31 antibody has been validated by Absin in western blot analysis of RAB31 expression in MCF7, Raw264.7, and rat brain whole cell lysates. Anti-VPS26B antibody has been validated by Absin in western blot analysis of protein expression in MCF7, Hela, PC12, AML12 whole cell lysates. Anti G3BP antibody has been validated by BD bioscience in immunofluorescence staining of A431 cells. It was also validated by plenty of publications. (https://www.bdbiosciences.com/zh-cn/products/reagents/microscopy-imaging-reagents/immunofluorescence-reagents/purified-mouse-anti-human-g3bp.611126).Anti-EDC4 antibody has been validated by Santa Cruz Biotechnology in immunofluorescence staining in methanol-fixed Hela cells. It was also validated by plenty of publications. (https://www.scbt.com/zh/p/edc4-antibody-h-12?requestFrom=search#citations)

Eukaryotic cell lines

Policy information about cell lines and Sex and Gender in Research

Cell line source(s)	Hela cells were purchased from cell bank of the Chinese Academy of Science, Shanghai, China.
Authentication	Hela cells have been authenticated by STR method.
Mycoplasma contamination	The cell line is mycoplasma negative.
Commonly misidentified lines<br>(See ICLAC register)	No commonly misidentified cell lines were used.
