# Supplementary material for: Machine learning predictor PSPire screens for phase-separating proteins lacking intrinsically disordered regions
Source: Nat Commun. 2024 Mar 8;15:2147. doi: 10.1038/s41467-024-46445-y (PMC10923898; doi:10.1038/s41467-024-46445-y)
Supplement: Supplementary file 3 — Description of Additional Supplementary Files [file 41467_2024_46445_MOESM3_ESM.pdf]

Title: Supplementary Data 1:
Description: Features used to train the PSPire classifier.

Title: Supplementary Data 2:
Description: Phase separation scores of human proteins predicted by PSPire.

Title: Supplementary Data 3:
Description: Highly and moderately confident PSP candidates.

Title: Supplementary Data 4:
Description: List of proteins in training and testing datasets.

Title: Supplementary Data 5:
Description: List of proteins in human MLO datasets.
